# Filopodia and Viruses: An Analysis of Membrane Processes in Entry Mechanisms

**DOI:** 10.3389/fmicb.2016.00300

**Published:** 2016-03-10

**Authors:** Kenneth Chang, John Baginski, Samer F. Hassan, Michael Volin, Deepak Shukla, Vaibhav Tiwari

**Affiliations:** ^1^Department of Microbiology and Immunology, Chicago College of Osteopathic Medicine, Midwestern UniversityDowners Grove, IL, USA; ^2^Department of Ophthalmology and Visual Sciences, University of Illinois at ChicagoChicago, IL, USA

**Keywords:** filopodia, heparan sulfate, virus-cell interaction, virus entry

## Abstract

Filopodia are thin, actin rich bundles protruding from cell plasma membranes, serving physiological purposes, such as probing the environment and facilitating cell-to-cell adhesion. Recent studies have highlighted that actively polymerized filopodial-protrusions are exploited during virus entry, trafficking, spread, and the development of clinical pathology of viral diseases. These observations have caused a surge in investigation of the key determinants of filopodial induction and their influence on cell topography including receptor expression for viral entry. It is now very clear that filopodia can provide unique opportunities for many viruses to invade host cells vertically during primary infection, or horizontally during virus spread from cell-to-cell. These emerging concepts can explain the unprecedented ability of viruses to invade both nearby and long-distant host cells, a feature that may directly contribute to viral tropism. In this review, we summarize the significance of filopodia in viral diseases and discuss future therapeutic possibilities to precisely target filopodial-flyovers to prevent or control infectious diseases.

## Introduction: Filopodia- A Master Explorer

Filopodia are actin-rich plasma-membrane processes that allow cells to probe their environment (Mattila and Lappalainen, 2008). The functions of filopodia are broad in nature; they contribute to wound healing processes, adhesion to the extracellular matrix (ECM), guidance toward chemoattractants, neuronal growth-cone path finding and embryonic development. Their size can range from 10 μm in length (nerve growth-cone filopodia) to 40 μm in length (sea-urchin embryo filopodia) (Welch and Mullins, 2002). Filopodia, or microspikes, may also be present in the cell cortex or leading edge. Despite the vital role of filopodia in cell functions, the biological mechanisms that govern filopodial functions are not completely understood. Current studies have only scratched the surface of the numerous roles of filopodia, whether beneficial or pathogenic. An emerging example of filopodia functions in infectious diseases is the recent discovery of the role of filopodia in viral surfing during entry and trafficking (Lehmann et al., 2005; Dixit et al., 2008; Oh et al., 2010; Bienkowska-Haba and Sapp, 2011; Schudt et al., 2013; Xu et al., 2015). Similarly, virus activated filopodia in dendritic cells have been proposed to transfer the virus with higher efficiency compared to the cell free route (Nikolic et al., 2011; Shrivastava et al., 2015).

One long-standing function of filopodia is to probe the environment and promote cell motility. It has receptors for signaling molecules and ECM molecules, which allows it to sense the surroundings and interact with other cells. Along the tip and shaft of filopodia exist two classes of cell adhesion molecules known as integrins and cadherins (Mattila and Lappalainen, 2008). During cell spreading, integrin-containing filopodia form the initial adhesion sites. Then, other components, such as talin and paxillin, are recruited to form the mature focal adhesions (Mattila and Lappalainen, 2008). Filopodia also play a role in cell-to-cell adhesion (Mattila and Lappalainen, 2008). An example of this interaction can be seen during embryonic development and wound healing (Mattila and Lappalainen, 2008). During these processes, there is a fusion of sheets of epithelial cells. Dynamic filopodia are present at the edges of epithelial cells and project to adjacent cells to aid in these processes. Interdigitated filopodia, which protrude from opposing cells, also help the sheets of cells align and adhere together (Mattila and Lappalainen, 2008). Embryonic epithelial fusion must occur in a precise fashion such as for dorsal closure in *Drosophila*. Filopodia facilitate cell-to-cell matching by allowing a cell to search for its match, and then pull misaligned sheets into alignment (Mattila and Lappalainen, 2008).

Membrane protrusions from neurons have been the most extensively studied (Bornschlogl, 2013). Filopodia are active in the neuronal growth cones of neurons, which guide axons and dendrites to their proper targets (Dent et al., 2011). Growth cones contain many filopodia. In this instance, filopodia sense the various gradients of chemoattractants and guide the neurite in the appropriate direction (Mattila and Lappalainen, 2008). However, filopodia are not essential for all types of neurite guidance. Retinal ganglion cells that are depleted of filopodia can still migrate along the optic tract, but they fail to establish axon terminal branching inside the tectum. The tectum is the midbrain “roof” and receives information from the retina (Mattila and Lappalainen, 2008).

## Structural Elements of Filopodia

High-resolution electron microscopy has shown that the core of filopodia consists of 15-30 tightly packed actin filaments, with a dense protein complex at the tip (Bornschlogl, 2013). The actin filaments in filopodia are cross-linked by fascin into a stiff structure (Leijnse et al., 2015a). There are proteins, which maintain the shape of the membrane, such as I-BAR proteins, which are actin-cytoskeleton adaptors that have been shown to contribute to formation of filopodia (Chen et al., 2015). Integrins are the transmembrane proteins, which link the actin to extracellular substrates to transfer force onto the substrate (Takada et al., 2007). Myosin-X, which provides a link between integrins and the cytoskeleton, transports membrane components like integrins to the filopodial tip (Zhang et al., 2004; Leijnse et al., 2015a). Other proteins include Enabled/vasodilator-stimulated phosphoprotein (Ena/VASP) and cofilin. There are high concentrations of fascin within the actin bundle (Leijnse et al., 2015a). Fascin are important because they are present in metastatic cancer cells as a notable hallmark. The role of filopodia in cancer is described at the end of this review article. In addition, protruding and retracting filopodia differ in actin composition. Protruding filopodia have a continuous actin filament, while retracting filopodia contain more disorganized and discontinuous actin filaments (Leijnse et al., 2015a).

Actin fibers present in filopodia have two forms: monomeric globular actin, also known as G-actin and polymeric filamentous actin, or F-actin (Cooper, 2000). F-actin is composed of two parallel strands of actin monomers. During viral infection, cellular actin is reconfigured and reorganized to affect the different stages of the viral life cycle. Polymerization of actin monomers begins with stabilization by an initiation complex. An example of this is the actin-related protein-2/3 (ARP2/3) complex. ARP2/3 is an initiation complex that is known to interact with viruses and multiple other pathogens (Cossart, 2000; Komano et al., 2004; Taylor et al., 2011). Taken togther, cells use a large repertoire of proteins to remodel the actin cytoskeleton including many events of simultaneous breakdown and assembly asprofilin can stimulate F-actin assembly, while cofilin promotes disassembly (Taylor et al., 2011).

F-actin is a part of the cytoskeleton composed of two parallel strands of ATP-bound globular actin monomers, which can assemble into finger-like protrusions (Xiang et al., 2012). The assembly of actin is involved in neuronal morphogenesis and migration. Errors in actin assembly within the neuron can lead to neurological diseases, such as non-syndromic X-linked mental retardation and William’s syndrome (Xiang et al., 2012).

Actin molecule binds to small proteins that help control its polymerization (Alberts et al., 1994). For example, the actin-depolymerizing factor (ADF)/cofilin family remodels the actin cytoskeleton. Cofilin is a family of actin-binding proteins, which disassemble F-actin by binding to older ADP-actin filaments and promoting phosphate disassociation from actin subunits (Xiang et al., 2012).

Polymerization of actin is critical to filopodial mechanics. To increase the length of the filopodia, the filopodial actin core grows inside the plasma membrane tube and then pushes out. Force is required because the plasma membrane is constantly under tension. It is the polymerization of actin that can produce the necessary protrusion force, often against 10 pN (piconewton) (Bornschlogl, 2013). Within the cell, actin is also arranged in sheet-like extensions, such as lamellipodia, microvilli, podosomes, and large membrane ruﬄes (Taylor et al., 2011). The length of filopodia ranges from 1 to 100 μm. Short filopodia are often called microspikes, while longer ones are called cytonemes or tunneling nanotubes (TNTs). Cytonemes can form long distance intercellular bridges. (Leijnse et al., 2015a). In addition, greater TNT production has been observed in the presence of HIV-1 (Hashimoto et al., 2016). Another type of cellular protrusion is known as lamellipodia, which are thin and sheet-like with a branched network of actin. Filopodia often protrude from the lamellipodial actin network as thin finger-like structures filled with tight parallel bundles of filamentous F-actin. In order for the cell to migrate, actin filaments push the leading edge (front of the cell) forward. (Mattila and Lappalainen, 2008).

Another role of filopodia is the initial neurite formation of cortical neurons. Dendritic spines are the postsynaptic regions of most excitatory neuronal synapses. These spines have been shown to play a role in higher brain functions, such as learning and memory. Dendritic spines require filopodia-like precursors. (Mattila and Lappalainen, 2008). Neurites can provide unique sites for virus adhesion and eventual spread to nearby cells and other neurons.

## Viral Utilization of Filopodia

Enveloped viruses can spread by two different routes: the cell-free aqueous environment or by cell-to-cell contact (Sherer et al., 2007; Mothes et al., 2010). Cell-free spreading requires a large number of viral particles to be released and to reach distant areas by diffusion. If these criteria are not met, cell-free spreading is impaired. Furthermore, cell-free virus is advantageous because it is not restricted to cell-to-cell interaction and can spread from person to person (Mothes et al., 2010). In contrast, cell-to-cell contact has unique advantages. The speed of transfer is greater because the replication cycle of release, transmission, and entry can proceed in a faster manner. Another advantage of cell-to-cell contact is immune evasion because the limited exposure in extracellular space avoids interaction with neutralizing antibodies. Lastly, by exploiting cell-to-cell communication, the physical and immunological barriers can be overcome to spread the infection (Mothes et al., 2010).

**FIGURE 1:**
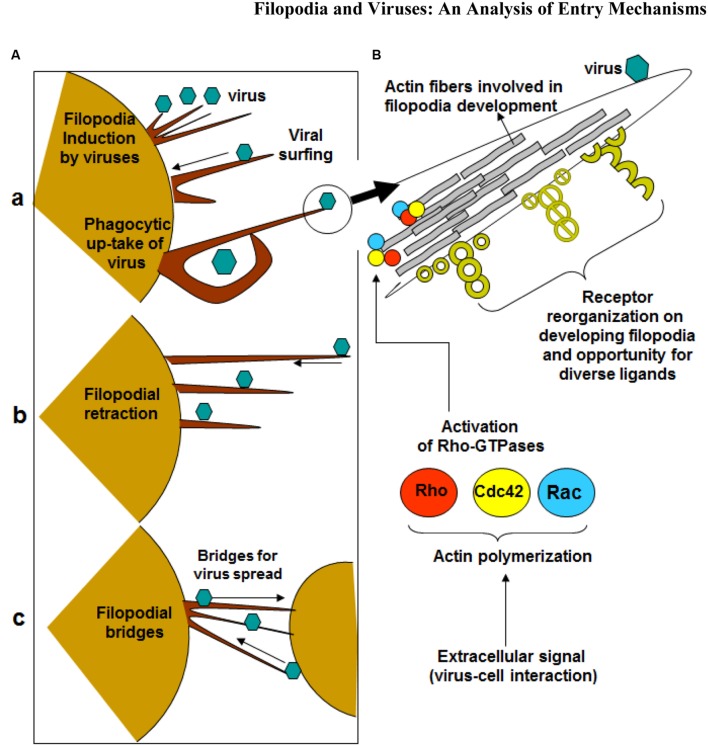
**Significance of filopodia in viral entry and cell-to-cell spread. (A)** Diagrammatic presentation of (a) virus induced filopodia, virus surfing on filopodia to reach cell body, (b) filopodial retractions, (c) and filopodial bridges to assist virus transport between the cells. **(B)** Virus-cell interactions activates Rho-GTPases (Rho, cdc42 and Rac) which potentially regulates actin assembly, filopodia formation, cell polarity, stress fibers, and filopodia associated receptors which assists in virus infection of the host cell.

Viruses can hijack the filopodia system for their own use during the life cycle (**Figure 1**) (Tiwari et al., 2015). Viral infection, a battle between pathogen and host, can occur because the virus travels along the filopodia from the infected cell toward the target cell. The ensuing result is “cell death, elimination of the virus, or latent infection, keeping both cells and pathogens alive” (Greber, 2002). It should be noted that pathogens and immune cells both could utilize filopodia in the different ways. For example, invasive bacteria use the protrusions of epithelial cells to approach the host cell before infection occurs, and macrophages use them as precursors for phagocytosis and their filopodia can capture both latex beads and beads covered with bacterial surface proteins (Bornschlogl, 2013). Viruses can travel along filopodia to enter the target cell. In addition to providing entry to an uninfected (target) cell, the filopodia can be used as an exit from an infected cell. In addition, host enzymes can induce filopodia formation upon sensing viral invasion. It was shown that zebrafish encoded heparan sulfate (HS) modifying enzyme 3-O sulfotransferase-3 (3-OST-3), which also generates a HSV-1 entry receptor, enhances filopodial protrusion formation and promotes viral entry (Choudhary et al., 2013). Given that viruses take advantage of filopodia during their life cycle, therapeutics can target filopodial formation to inhibit infection.

## G-Protein Stimulation in Filopodia Formation

Small GTPases of the Rho superfamily regulate cell morphology, particularly the actin cytoskeleton. Members of this family regulate many cellular processes, including F-actin polymerization, assembly of intercellular junctions, cell polarity, and membrane trafficking. Pathogens, such as viruses, possess gene products that engage and disrupt the actin cytoskeleton and Rho-family GTPase signaling system (**Figure 1**). Ultimately, F-actin is remodeled for the main stages of the viral life cycle - entry, assembly, and egress (Taylor et al., 2011). The best-studied mammalian Rho GTPases are RhoA, Cdc42, and Rac1. RhoA stimulates the formation of stress fibers while Rac1 induces membrane ruﬄes or lamellipodia. Cdc42 regulates the formation of filopodia (Taylor et al., 2011). It operates through distinct signaling pathways, such as the induction of the ARP2/3 complex-dependent actin filament nucleation through activation of Wiskott–Aldrich syndrome protein (WASP) (Mattila and Lappalainen, 2008). ARP2/3 complex initiates polymerization of actin monomers; it is the complex most often described as interacting with viruses. Although ARP2/3 has little polymerization-stimulating activity, interaction with induction factors such as the WASP will enhance polymerization ability (Taylor et al., 2011).

Although Cdc42 is important, filopodia formation can occur in cells depleted of Cdc42. A small GTPase, called Rho in filopodia (RIF) can stimulate filopodia formation following overexpression. In contrast to the filopodia induced by Cdc42, the filopodia induced by RIF are longer and project from the apical surface of the cell. Overall, multiple Rho GTPases can induce cellular protrusion when over-expressed, but their roles under physiological conditions remain to be explained (Mattila and Lappalainen, 2008).

### Cdc-42 – A Model Rho GTPase Supporting Viral Invasion

Cdc42 is a RhoA GTPase that has been associated with cell mediated processes involving actin modifications. Rho-GTPases are associated with events such as modulation of cytoskeletal components, cell migration, cell trafficking and cell polarity (Nikolic et al., 2011). Numerous pathogens, both bacterial and viral, have been shown to interact with Cdc42 within the host cell to facilitate the remodeling of actin within the host cells. In the cases of viruses such as herpes simplex virus-1 (HSV-1) and human immunodeficiency virus (HIV), viral activation of Cdc42 leads to the production of filopodia and filopodia-like structures. Studies performed with HSV-1 have demonstrated that a down-regulation of Cdc42 not only results in a decline in filopodia but also infection in general (Oh et al., 2010). Similar studies performed with HIV demonstrated similar results when Cdc42 was inhibited with secramine A, indicating the potential role of Cdc42 in viral infections (Nikolic et al., 2011). Guillou et al. (2008) performed an experiment wherein fibroblasts spread on micropatterned surfaces. Filopodia, which contains adhesion structures, converted to lamellipodia-like protrusions. Expression of a dominant-negative form of the small GTPase Cdc42 stopped filopodia formation, which impaired cell spreading (Mattila and Lappalainen, 2008).

It has been postulated that viral activation of Cdc42 is mediated by Src kinases. Src kinase is a non-receptor tyrosine kinase protein (Wheeler et al., 2009). In a study performed by Nikolic et al. (2011) incubation of dendritic cells with wild type HIV, demonstrated activation of Src kinase, Pak1, and WASP. Inhibition of Cdc42 or the Src kinases suggested that the initial Src activation occurs upstream of Cdc42, Pak1 and WASP. Nikolic suggests Src kinases serve as a link between HIV interaction on the surface of the cell with the subsequent activation of Cdc42, Pak1, and WASP, leading to the eventual increase in cell surface extensions (Nikolic et al., 2011). Levels of Src kinase are linked to cancer progression by promoting other signals (Wheeler et al., 2009). Therefore, since Cdc42 pathway is mediated by Src kinase, there is a link between cancer and filopodia formation (Cancer – Src kinase – Cdc42 – Filopodia). This linkage is another evidence of how filopodia promotes cancer formation as described at the end of this review article.

### Rho Proteins Involvement in HSV-1 Infection

It has been speculated that RhoA, a member of the Rho GTPase family, may play a role in HSV-1 infection. Studies have suggested that RhoA may facilitate entry of HSV-1 via endocytosis into human corneal fibroblasts (Salameh et al., 2012). Other members of the Rho GTPase family have been implicated in the development of filopodia (Salameh et al., 2012). Specifically, Rac-1 and CDC42 are activated in epithelial cells and fibroblasts during HSV-1 infection (Quetglas et al., 2012). To complicate matters, signaling pathways change for different cells. In nectin-1-CHO and corneal fibroblasts, RhoA is induced, but not Cdc42 or Rac1 (Clement et al., 2006). The different mechanisms of communication may explain why the HSV-1 mode of entry is cell type-specific. For example, filopodia play a role in HSV-1 entry for nectin-1-CHO cells. However, in cells from human trabecular meshwork tissues, relatively few protrusions are seen and intracellular vesicles containing virions were absent (Clement et al., 2006).

## Overview of Viral Entry Mechanisms

Filopodia provide a way for cells to physically interact with each other and transmit materials such as ligands or other substances. Retroviruses exploit the transport mechanism, thereby facilitating infection by movement of viruses and viral proteins (Sherer et al., 2007). Inhibiting the surfing mechanism results in a modest reduction in infection of cultured fibroblast cells where viruses have relatively easy access to the cell body. However, interference with surfing leads to a significant reduction in infection of polarized epithelial cells that are covered in dense microvilli. Lehmann et al. (2005) hypothesize that virus cell surfing enables the penetration of the microvilli-rich mucosal surfaces to efficiently infect the host. An example of mucosal transmission is HIV infection, through which mucosal transmission occurs for 80% of infections (Lehmann et al., 2005).

Specialized movements occur among different viruses. For example, animal viruses typically display a period of lateral movement along filopodia before internalizing into the cell. A highly directional movement exists for murine leukemia virus (MLV), enveloped RNA viruses, and human papilloma virus-16 (HPV-16). HPV-16 is linked to the development of cervical cancer (Schelhaas et al., 2008). In addition, filopodia can be a marker for viral infection. The number of these actin-rich protrusions increases during infection with either HSV-1, cytomegalovirus (CMV), or human herpesvirus-8 (HHV-8). Experiments were performed on three different cell types for each virus. The number of filopodia increased in varying degrees, from 2-fold increase to 6-fold increase (Tiwari and Shukla, 2012).

Lastly, filopodia provide an alternative mechanism for viral entry. In vaccinia virus (VACV), the two strains – International Health Department-J (IHD-J) and Western Reserve (WR) – enter host cells by different mechanisms. IHD-J mature virions (MVs) induce filopodia formation, such that exposure to IHD-J increases the number and length of filopodia dramatically. Filopodia remain a permanent feature of the infected cells for many hours. In contrast, WR MVs alter actin dynamics, which leads to transient membrane blebbing followed by macropinocytic internalization of virus particles. Unlike the filopodia of IHD-J, the blebs induced by WR MVs are transient (Mercer et al., 2010a).

### Viral Binding

Glycoproteins play an important role in viral attachment to cells. Their positively charged moieties often recognize negatively charged domains of cell surface receptors such as HS. As number of human viruses including HSV-1, HHV-8, HPV-16, HIV, avian sarcoma leukosis virus (ALV), and vesicular stomatitis virus (VSV) bind HS (Tiwari et al., 2012), and the latter is shown to be highly expressed on filopodia (Oh et al., 2010), it is likely that filopodia contribute directly to virus binding. In fact, it has been clearly shown with HSV-1 that its preferred binding location on the cell surface is filopodia (Oh et al., 2010). The HSV glycoprotein that participates in filopodial binding is gB, which is a known HS binding protein. Although remains to be shown, it is likely that for other viruses their HS binding proteins are responsible for viral interactions with filopodia.

### Viral Surfing

Binding of virus to filopodia induced a rapid lateral movement toward the cell body, a process called surfing (Lehmann et al., 2005). Viral Surfing is a mechanism through which high affinity interactions with receptor on the target cell engage the F-actin flow to move toward the cell body of the target cell (Mothes et al., 2010) (**Figure 1**). Filopodia have been proven to be involved in the surfing process, as well as in the process of inhibition of surfing. Since virus cell surfing is a pathway of viral infection, the visual block to surfing by blebbistatin and cytochalasin D theoretically should result in reduced infectivity. Studies looking at the earlier stages of infection found that the inhibitory effects of blebbistatin on MLV and VSV were significant only when cells were cultured at low confluencies. Under these conditions, cells exhibit abundant filopodia. At higher confluencies, when cells contacted each other, blebbistatin had reduced effects. This suggests that blebbistatin affects the earliest steps of MLV entry when cells exhibit abundant filopodia (Lehmann et al., 2005). Similarly, cytochalasin D had greater inhibitory effects on virus entry when cells contained abundant filopodia.

Mucosal epithelia are also relevant to viral infection. Polarized epithelial cells covered with dense microvilli (MDCK cells) have been tested to see if surfing was required for efficient infection. The data obtained indicated that infection of polarized MDCK cells by MLV or VSVG-containing viruses was reduced by at least fivefold in the presence blebbistatin and cytochalasin D when virus was added to the microvilli-rich apical side but not to the basolateral side. Blebbistatin and cytochalasin D were added in separate treatments (Lehmann et al., 2005). In comparing infection of cultured fibroblast cells to infection of polarized epithelial cells, only a modest reduction in infection of cultured fibroblast cells was observed. It has been shown that viruses have relatively easy access to the fibroblast cell body (Lehmann et al., 2005). However, a blockade to surfing leads to a reduction in infection of polarized epithelial cells. Lehmann et al. (2005) hypothesized that *in vivo*, virus cell surfing enables the penetration of microvilli-rich mucosal surfaces to efficiently infect the host.

### Filopodial Retraction

The filopodia on virally infected cells pull the virus inward (**Figure 1**). A similar action is seen when macrophages bind to bacteria and pull them toward the cell body, where phagocytosis occurs. Macrophages utilize numerous filopodia per cell to explore the environment. When a pathogen is found, the filopodia bind to it and retract toward the macrophage cell body (Mattila and Lappalainen, 2008). The pulling mechanism is conserved because cells retract filopodia for entry of certain virus, such as HPV (Smith et al., 2008).

A hypothesized model for how the filopodia pulls is through an action known as “frictional coupling” (Bornschlogl, 2013). Since filopodia and lamellipodial structures are connected, friction could drive retrograde flow (Bornschlogl, 2013). The cytoskeleton is generating friction on the substrate environment. Filopodia act along with lamellipodial actin network to drive retrograde flow. The cytoskeleton was shown to promote retraction force rather than just membrane tension by the finding that the force was measured at 1-2 nN (Bornschlogl, 2013).

The retraction force was examined through an experimental set-up consisting of a bead attached to filopodia and observation through confocal fluorescence microscopy (Bornschlogl, 2013). The extension and retraction of filopodia was determined by the difference between the actin polymerization rate at the tip and the retrograde flow at the base of the filopodium. Actin polymerization was shown to reduce locally at the tip and then the protrusion retracted. Within this model, the bead is analogous to the virus. The results obtained allow for easy data collection regarding the actual retraction forces. (Bornsclogl et al., 2013).

Originally, the pulling force of filopodia was attributed to retrograde flow of actin, but recently, experiments show that rotational dynamics also make a contribution. Force measurements indicate a step-like behavior for the retraction force, but no direct evidence links the behavior to the myosin motor protein. Thus, the mechanical and biochemical mechanism for filopodial retraction requires further examination (Leijnse et al., 2015a).

### Endocytosis of Virus

Conversely, after viruses flow along filopodia, they can undergo endocytosis to enter the uninfected cell. Most animal viruses depend on endocytic uptake, vesicular transport through the cytoplasm, and delivery to endosomes and other intracellular organelles. The internalization may involve clathrin-mediated endocytosis (CME), macropinocytosis, caveolar/lipid raft-mediated endocytosis, or a variety of other still poorly characterized mechanisms (Mercer et al., 2010b). It should be noted that viruses could skip the filopodia transport (**Figure 1**). In this instance, they would simply bind to receptors on the cell surface and induce endocytosis. Receptors promote entry “by binding, by initiating conformational changes in the virus, by activating cellular signaling, and by inducing fusion at the plasma membrane or by promoting endocytosis” (Schelhaas, 2010). Both fusion and endocytosis can be utilized, but the latter is advantageous for three reasons. First, there will be a delay in immune response because there is no trace of virus on the plasma membrane. Second, the built-in transport mechanism across the plasma membrane is utilized. Third, vesicular trafficking provides access to intracellular organelles, allowing virus to sense their environment to changing conditions of pH, redox, and proteases (Schelhaas, 2010). Most RNA viruses replicate in the cytosol, while DNA viruses must gain access to the nucleus to facilitate this process (Schelhaas, 2010). Endocytosis is important for eukaryotic cells to internalize extracellular particles, fluid and ligands. The process begins with the formation of primary endocytic vesicles, which pinch-off from the plasma membrane. It has been long thought that receptor-mediated endocytosis consists mainly of clathrine-mediated endocytosis (Schelhaas, 2010). Now, recent research shows additional pathways: macropinocytosis, caveolar/raft-dependent endocytosis, among others (Schelhaas, 2010). Taken together, different viruses use different endocytic pathways.

### Viral Budding

Filopodia have been discussed above as a structural component for viral entry. However, filopodia can also be used to facilitate “budding”- a common exit mechanism for viruses. For example, Marburg virus (MARV) and Ebola virus virogenesis begins with formation of perinuclear inclusions which are sites of viral replication and assembly of new nucleocapsids (NCs). NCs are detected in the cytosol, at the plasma membrane, and in filopodia, the preferred sites of MARV budding (Schudt et al., 2013). In case of Varicella-Zoster virus and Sindbis virus, the imaging studies have demonstrated the presence of virions on filopodia during viral egress (Carpenter et al., 2008; Jose et al., 2015).

The actin cortex can facilitate or hinder the movement of virus that bud at the plasma membrane. For VACV, actin polymerization helps propel the virus away. For equine infectious anemia virus, actin cortex hindered the release of viral particles (Kolesnikova et al., 2007). Therefore, budding involves a rearrangement of the actin cortex. Once the virus buds, the presence of actin in purified virus particles is a sign that the process has occurred (Kolesnikova et al., 2007). Filopodial budding is advantageous because filopodia make connections to neighboring cells, thus facilitating access to the next target cell. On the other hand, the release of budding virions directly to target cells minimizes the appearance of virions in the extracellular space, which avoids recognition by the hostile environment (immune system) (Schudt et al., 2013).

Observations of mouse mammary tumor virus (MMTV), a retrovirus, revealed the first connection between the virion exit and the actin cytoskeleton (Taylor et al., 2011). Mammary tumor epithelial cells released viral particles from the ends of microvilli (Taylor et al., 2011). Since HIV is also a retrovirus, it was important to determine whether viral proteins interact with the actin cytoskeleton for the final steps of assembly and release. Studies in which actin filaments were disrupted by cytochalasin D lead to an impairment of virus budding and reduced virus yield by 80% (Taylor et al., 2011).

## Cytoskeletal Rearrangement

Virus-induced cytoskeletal rearrangements are commonly initiated via cellular signaling to prepare and facilitate infection. Rearrangement of cortical actin filaments is observed in virus infected cells and has been shown contribute to infection efficiency. Once internalized, the virus can use microtubule cytoskeleton and motor protein machinery to reach the nucleus (Smith et al., 2008).

Different findings have been reported about the mechanisms of actin assembly in filopodia. The convergent elongation model argues that “filopodia arise from the lamellipodial F-actin network and a continuous actin bundle extends from the root to the tip of filopodia” (Mattila and Lappalainen, 2008). The *de novo* filament nucleation model states that actin filaments of filopodia “do not derive from the underlying lamellipodial network, but are nucleated at filopodial tips by formins” (Mattila and Lappalainen, 2008).

## Examples of Viral Interaction with Filopodia

### HSV-1

**FIGURE 2:**
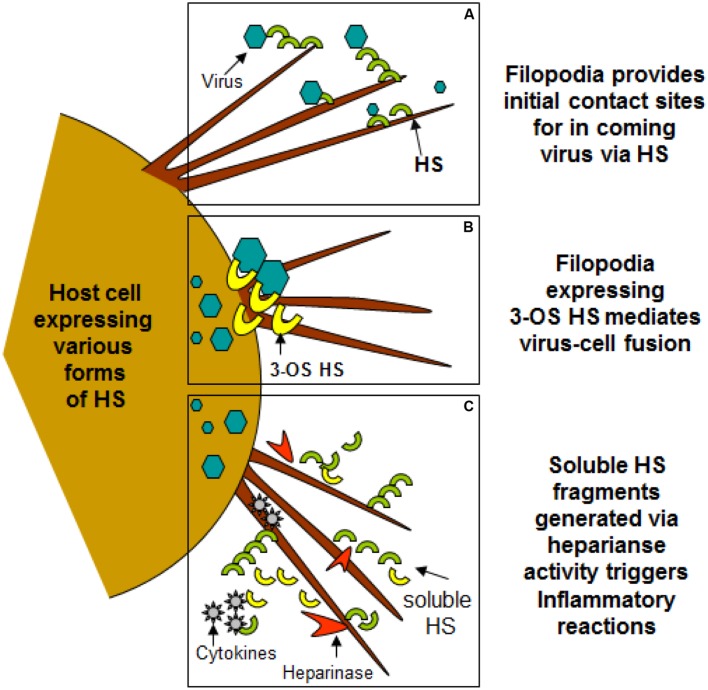
**Filopodia expresses diverse form of heparan sulfate (HS) and 3-*O* sulfated heparan sulfate (3-*O*S HS) assisting in viral entry of HSV-1. (A,B)** Shows HS and 3-*O*S HS in assisting virus attachment and fusion events using filopodia. **(C)** Indicates the upregulation of heparinase activity during infection results shedding of HS/3-*O*S HS which potentially are involved in recruitment of inflammatory cells which may utilize filopodia.

(HSV-1) and herpes simplex virus-2 (HSV-2) are some of the first viruses to have demonstrated a dependency upon filopodia during infection. They are part of the herpesviridae family, which consists of over 70 viral species: including varicella-zoster virus, CMV, human herpesvirus-6 (HHV-6) and Epstein-Barr virus. Herpesviruses have linear, double-stranded DNA enclosed in icosahedral capsids. They enter latency after primary infection, establishing infection for the lifetime of their hosts (Salameh et al., 2012). During stressful conditions, HSV-1 reactivates and proceeds with viral replication, leading to perioral lesions of the skin, mucosa, or lesions on the cornea. On the other hand, HSV-2 is primarily associated with genital and newborn infections (Xiang et al., 2012). HSV-1 has been shown to travel down filopodia-like membrane protrusions to reach the cell body for internalization. This action appears to be regulated by activation of Cdc42 (Oh et al., 2010). Exposure to HSV-1 can induce the formation of actin-rich, filopodia-like structures by the cell. Filopodial formation is facilitated through members of the Rho GTPase family, which serve as a link between surface receptors and the actin cytoskeleton underneath. Glycoprotein gB seems to regulate viral surfing. This notion is reinforced by the fact that gB binds to HS (Oh et al., 2010). HS receptors serve as attachment sites for HSV-1 which is also present on filopodia (**Figure 2**). Once the virus binds, it can travel to the cell surface, where gD proceeds to bind with one of its four receptors. The process of virus penetration and membrane fusion follows (Salameh et al., 2012).

#### Entry Mechanisms

Heparan sulfate glycosaminoglycans are hybrid molecules with unbranched polysaccharide polymers covalently attached to the protein core. HS can be sulfated to produce 3-*O*-sulfated HS which can independently make cells susceptible to HSV infection. Glycoprotein B and C are bound first to unmodified HS; next, gD binds to 3-*O*S HS to facilitate fusion pore formation during viral entry. Again presence of 3-*O*S HS on filopodia may allow virus-cell fusion (**Figure 2**). Virus trafficking via virus-cell fusion at the cell surface results in the release of viral genome into the nucleus. Deconvolution microscopy using capsid-tagged green fluorescent virus showed that HSV-1 can infect the corneal stroma through HS associated trafficking (Tiwari et al., 2015).

#### Endocytosis

HSV-1 can induce primary human corneal fibroblasts to engulf it in a form of an atypical endocytosis (Clement et al., 2006). The uptake of HSV resembles phagocytosis because it is not pH dependent and does not involve the use of clathrin-coated vesicles or caveolae (Salameh et al., 2012). Furthermore, there is rearrangement of actin cytoskeleton and trafficking of the virions in large phagosome-like vesicles. HSV-1 can induce endocytosis of *E. coli* bioparticles and virions cointernalized with phagocytic tracers (Clement et al., 2006). Endocytosis would be the second method through which HSV-1 can enter cells, with the first being surfing. Transport is initially along filopodia and virion fusion occurs at the vesicular membrane.

#### Cytoskeletal Rearrangements

HSV interacts with the host cytoskeleton, specifically with the F-actin components. A role for cofilin was discovered in HSV-1 infection. HSV-1 infection increases F-actin assembly at the early stage of infection to facilitate viral transport. In the later stages of infection, F-actin decreases to facilitate viral reproduction. Therefore, HSV-1 infection induces biphasic dynamics of F-actin in neuroblastoma cells (Xiang et al., 2012). Cofilin-1 regulation may mediate HSV-1-induced F-actin remodeling in assembly and disassembly. Specifically, Cofilin-1 may promote F-actin assembly during the HSV-1 infection of neuronal cells. Regulation of Cofilin-1 decreased the formation of F-actin-based structures, such as lamellipodia (Xiang et al., 2012).

F-actin is important for HSV-1 infection. In the past, the major capsid protein of HSV-1 has been immunostained and utilized as a marker to indicate localization of HSV-1 particles. Cells infected by HSV-1 have been shown to grow long dendrites and filopodia. The filopodia formed during this infection have been found to have viral particles “docked” on them (Xiang et al., 2012). This suggests that HSV-1 may interact with F-actin for transport to the soma. The viral particles were randomly distributed around the cell and approached the soma and nucleus from many directions. With cytoskeletal rearrangement involving F-actin, HSV-1 can infect the cell by interacting with F-actin (Xiang et al., 2012).

### HPV – The Ultimate Filopodial Utilization

In a study performed by Smith et al. (2008), filopodial structures facilitate viral uptake because induction of filopodia occurred at 30 min after binding. Over several hours, the virions began to disappear from the ECM. Viral diffusion was ruled out by fluorescence recovery after photobleaching (FRAP). The results from FRAP indicated that the virions were immobile. It was later revealed that filopodia can act to increase virion acquisition from the ECM to cells over many hours. The HPV31 virions have a long internalization half time (14 h) because of the active and protracted virion uptake from the ECM by filopodia (Smith et al., 2008).

Other direct evidence showed that HPV surfs along filopodia. Electron microscopy and acid wash of FITC-labeled virions also showed the association between virions and the outer surface of filopodia. Using fluorescence video microscopy, the human papilloma virus-16 (HPV-16) virion demonstrated a transport speed equal to the speed of F-actin retrograde flow. Inhibitors of actin polymerization and depolymerization affected HPV-16 directed flow, which again suggests transport via actin. (Bienkowska-Haba and Sapp, 2011). It was notable that transport along actin protrusions significantly enhanced HPV-16 infection in sparse tissue culture cells, but not in dense cultures (Schelhaas et al., 2008).

Studies using human papilloma virus-31 (HPV-31) have been the first to report filopodial translocation by a non-enveloped virus (Smith et al., 2008). The retrograde filopodial movement and entry of HPV-31 are comparable to those seen for MLV and VSV infections. It is interesting to note that a few HPV-31 virions bind directly to the plasma membrane where they enter prior to filopodia activation in human keratinocytes (Hks). This argues against a strict requirement for filopodia in HPV31 infection of HKs. Rather, filopodial transport serves to increase cell exposure to virions and particle uptake (Smith et al., 2008).

#### Retraction and Lateral Curling

Aside from surfing, two other modes of distal particle transfer to the cell body via filopodia occur: filopodial retraction and lateral curling. Lateral curling is when the filopodial tip with attached virion bends back toward the cell body. (Smith et al., 2008). The lateral curling mechanism is significant because it indicates that another pulling mechanism exists to retract filopodia. Another name for lateral curling is buckling, which has been observed by confocal microscopy and atomic force spectroscopy. The rotation of the actin shaft accumulates torsional energy in the actin that can be released by helical bending. Studies examining the filopodia of human embryonic kidney-293 (HEK-293) cells revealed rotation, bending, and shortening of the actin within filopodia (Leijnse et al., 2015a). Buckling can be thought of as the “pulling and bending that arises when twisting a rubber band at one end and keeping the other end fixed” (Leijnse et al., 2015b).

#### Endocytosis

On the other hand, internalization of HPV is unusually slow and asynchronous, with half-times ranging from 4 and 14 h. HPV-16 is a lab strain and pseudovirion which carried a marker plasmid coding for a dimeric green fluorescent protein (GFP). HPV-16 internalizes via clathrin-, caveolin-, and dynamin-independent novel pathways. Studies using siRNAs and inhibitors of endocytosis excluded macropinocytosis and phagocytosis as possible mechanisms for internalization. (Bienkowska-Haba and Sapp, 2011). During observation of HPV-31, another lab strain, virion motion transitioned from active transport to rapid diffusion once the particles reached the cell body. This shift is indicative of one of two mechanisms: internalization at the base of the filopodia or transfer to the adherent (basolateral) cell surface (Smith et al., 2008). The mechanism for HPV-31 endocytosis is different from that of HPV-16. The reason for this difference is under debate. While HPV-31 endocytosis seems to occur via caveolae, one study showed that entry occurs through a clathrin-mediated pathway (Bienkowska-Haba and Sapp, 2011).

### Murine Leukemia Virus

For MLV, a group of retroviruses known to cause cancer in mice, the filopodia originates from the target cell and extends to the infected cell. The infected cell can exert a pull force to increase the length of viral cytonemes. Filopodial length can therefore increase toward the center of an infected cell. Extension is accompanied by a net flow of membrane toward the infected cell (Sherer et al., 2007). Specific effects of filopodia extension are discussed below.

#### Viral Invasion

For cells infected by MLV, the infected cell grabs filopodia using contacts between viral envelope glycoprotein (Env) and its receptor in the target cell. “The infected cell tears off and takes in chunks of filopodial membrane by endocytosis” (LeBrasseur, 2007). The tips of cytonemes colocalized at the infected cell surface with endocytic markers: dynamin 2, caveolin-1, cholera toxin B-subunit (Sherer et al., 2007). This allows invasion of the neighboring cell via viral transport on cytonemes.

#### Surfing Mechanism

Confocal time-lapse microscopy of HEK-293 cells showed that MLV viral particles attach to filopodia, but also underwent directed reverse movement, surfing toward the cell body. MLV labeled with a fusion-competent envelope-YFP protein (Env-YFP) was added to HEK 293 cells expressing the receptor mCAT-1 fused to CFP (mCAT-1-CFP). This assay demonstrated that when viral particles come in contact with the cell body of the mCAT-1-CFP expressing cells, the viral particles lose their envelope, as demonstrated by a loss of label. This result is consistent with the idea of fusion-mediated diffusion into the surface of the plasma membrane (Lehmann et al., 2005). The previously mentioned assay also demonstrated that surfing occurs prior viral fusion. This was indicated by the lack of complete diffusion of the Env-YFP label until reaching the base of the filopodia. Previous studies with an MLV carrying a fusion defective envelope demonstrated surfing characteristics. Fusion intermediates or intracellular capsids were detected only at the widening base of the filopodia or at the cell body. This suggests that the viral envelope is not required for surfing actions (Lehmann et al., 2005).

### Vaccinia Virus

The filopodia of HeLa cells infected with VACV have been shown to retract in two different ways. One is a ruﬄing retraction, where viruses bound to cell edges or active ruﬄing areas are transported to the cell body. The second way is ‘grabbing’ by the waving actin protrusions, which brings the virus back to the cell body (Huang et al., 2008).

#### Viral Entry

After binding to filopodia of HeLa cells, VACV enters cells through an endocytic route that requires a dynamin-mediated pathway, but not a clathrin- or caveola- mediated pathway. Virus entry involves a novel cellular protein, vaccinia virus penetration factor (VPEF) (Huang et al., 2008). VPEF was detected on lipid rafts in the plasma membrane and on vesicle-like structures in the cytoplasm (Huang et al., 2008). VPEF likely mediates VACV entry through a fluid uptake endocytosis process in HeLa cells. Huang et al. (2008) hypothesized that vaccinia intracellular MV recruited a cellular vesicle protein for its own benefit. Therefore, HeLa cells were incubated with fluorescence labeled ligands – dextran or transferrin. Dextran normally undergoes fluid phase endocytosis. In Huang’s experiment, dextran showed colocalization with VPEF. When VPEF expression was knocked down, vaccinia penetration and intracellular transport of dextran were both blocked. Due to these findings, VPEF plays a role in regulating the fluid phase of the endocytic pathway in HeLa cells (Huang et al., 2008).

#### Routes of Infection

In order to determine some of the differences and similarities between the various strains of VACV, some studies have focused on IHD-J and WR strains and their influences (Mercer et al., 2010a). As indicated previously, WR induces transient blebs on the surface of cells, while IHD-J induces permanent and lengthening filopodia. Aside from having two different strains, VACV is also notable for using two different routes of infection for HeLa cells and CHO cells (Mercer et al., 2010a). In HeLa cells, activation of epidermal growth factor receptor (EGFR) is a necessary step in infection. Blocking EGFR with antibodies and other agents inhibits VACV infection in L cells of the intestinal enteroendocrine cells. With CHO cells, VACV can infect the cell even though it does not express EGFR. Based on these results, it can be inferred VACV is part of a group of viruses that use alternative strategies to enter cells (Mercer et al., 2010b).

## Clinical Significance

**FIGURE 3:**
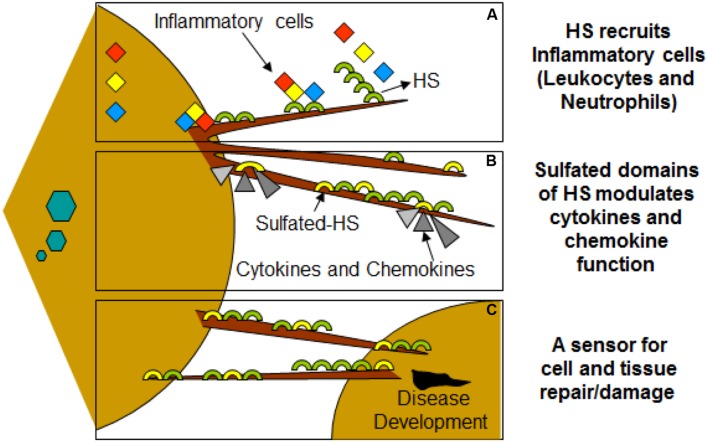
**Filopodia may play additional role in disease development during viral infection of HSV-1. (A)** Molecular diversity of HS present on filopodia results recruitments of leucocytes and neutrophils. **(B)** HS also assist in binding and bringing conformational changes in bound chemokines, establishing chemokine gradients. **(C)** HSV infected cells shed HS on the filopodia which may serve as a potent inducers of inflammatory response.

**FIGURE 4:**
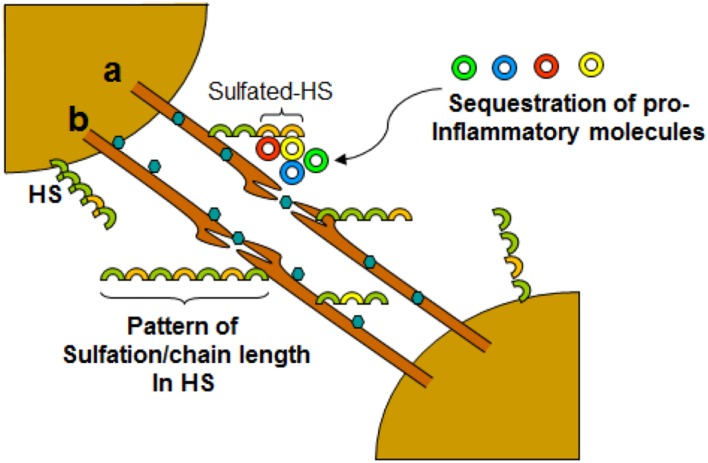
**Dynamic role of filopodia connecting neurons influencing HSV-1 infections and associated inflammation.** Virus travel either intracellular **(a)** or extracellular **(b)** between the cells. The diversity of HS chain in terms of sulfation pattern and chain length play a crucial role in interactions with cell surface molecules including leucocytes and soluble inflammatory cytokines.

Virus infection is also related to cancer because an oncovirus is a virus that can cause cancer (Butt and Miggin, 2012). For example, HPV can increase the risk of cervical cancer (American Cancer Society, 2015). HHV-8 is associated with Kaposi’s sarcoma, a type of skin cancer (Chang et al., 1994). Lastly, human cytamegalovirus is associated with mucoepidermoid carcinoma (Melnick et al., 2012). Therefore, a quick understanding of the use of filopodia by cancer cells shows the clinical importance of filopodia. The invasive motility in cancer cells is partly due to the formation of filopodia. Much attention has focused on the actin bundling protein, fascin, which localizes to filopodia, microspikes, and other actin-based protrusions underneath the plasma membrane (Machesky and Li, 2010). Fascin localizes to the leading edge of crawling cells and is important for assembly of filopodia. Inhibiting fascin results in reduced migration of the cell (Machesky and Li, 2010). Therefore, fascin provides normal and cancerous cells with increased motility. In aggressive and metastatic epithelial cancers, high fascin expression is an independent prognostic indicator of poor outcome (Machesky and Li, 2010). Cancers include: liver, ovary, lung, pancreas, colorectal, head and neck squamous cell carcinoma and brain (Machesky and Li, 2010). However, fascin expression is not applicable to many cancers, such as breast and prostate (Machesky and Li, 2010). For cancers such as biliary tract (gallbladder cancer), although fascin is not an independent prognostic indicator, it is marginally significant in correlating with poor prognosis (Machesky and Li, 2010). Further, filopodia or filopodial bridges may be able to trap the soluble fragments of diverse HS generated as results of high heparanase activity during HSV infection, which may facilitate influx of inflammatory cells (**Figure 2C**) (Hadigal et al., 2015). Similarly, specific forms of sulfated-HS on filopodia or on filopodial bridges may orchestrate wide variety of inflammatory reactions by regulating various inflammatory processes (**Figure 3**) (Simon Davis and Parish, 2013). Finally, HSV being a neurotropic virus widely utilizes neuronal axons to move across the cells. The later system expressing HS mimics filopodial model to influence virus infection and associated inflammation in the brain (**Figure 4**) (Zhang et al., 2014).

## Concluding Remarks

Filopodia were first identified for their roles in cell migration and chemoattractant sensory mechanisms through utilization of their neuronal growth cones. Recently, filopodia have gained increasing attention because they have been found to facilitate viral infection including viral spread (**Table 1**). Cell-to-cell spreading has advantages including increased speed, immune evasion, overcoming physiological barriers, and greater infectivity of cells. The process of viral entry using filopodia consists of multiple steps. Receptor and co-receptor binding leads to cellular signaling, which rearranges the cytoskeleton of the cell. Viruses are seen to ‘surf’ along filopodia, almost like beads on a conveyor belt. Filopodia further enable viral entry by filopodial retraction and endocytosis at the base of the filopodia. There are notable similarities among the viruses that utilize filopodia for viral entry. For example, HPV and HSV-1 surf along filopodia and internalize at the base of filopodia through endocytosis. Still, filopodia are so diverse in function that they can facilitate budding. Two prime examples are the related viruses, MARV and Ebola Virus. However, viral infection is not the only factor stimulating formation of these actin protrusions – inflammation also enhances filopodia. Inflammatory cytokines such as TNFα, IL-1β, INFγ and LPS produced filopodia in as early as 1 h. Second, chemokines, such as CXCL8, also facilitate filopodia formation. The filopodia were found to associate with leukocytes and facilitate increased neutrophil migration. Overall, filopodia are clinically significant not only because of its role in viral entry, exit, and inflammatory response, but also because filopodia promote cancer metastasis.

**Table 1:** Summary of cell mechanisms of filopodial entry by different viruses.

Virus entry Mechanisms	HSV-1^1^	HPV^2^	MLV^3^	ALV^4^	HIV-1^5^	vsv^6^	HHV-8^7^	MARV^8^	VACV^9^
Receptor/Co-receptor interaction	**^∗^**	**^∗^**	**^∗^**				**^∗^**		**^∗^**
Cytoskeletal rearrangements	**^∗^**	**^∗^**							**^∗^**
Surfing	**^∗^**	**^∗^**	**^∗^**	**^∗^**	**^∗^**	**^∗^**		**^∗^**	**^∗^**
Filopodia retraction		**^∗^**	**^∗^**						**^∗^**
Endocytosis	**^∗^**	**^∗^**	**^∗^**				**^∗^**		**^∗^**
Budding	**^∗^**		**^∗^**		**^∗^**			**^∗^**	**^∗^**
Alteration of entry mechanism	**^∗^**				**^∗^**				**^∗^**

*^1^HSV, herpes simplex virus: Clement et al., 2006; Taylor et al., 2011; Salameh et al., 2012; Xiang et al., 2012; Tiwari et al., 2015. ^2^HPV, human papilloma virus: Hindmarsh and Laimins, 2007; Schelhaas et al., 2008; Smith et al., 2008; Bienkowska-Haba and Sapp, 2011; Leijnse et al., 2015a,b). ^3^MLV, murine leukemia virus: Lehmann et al., 2005; Sherer et al., 2007; Mercer et al., 2010a. ^4^ALV, avian leukosis virus: Lehmann et al., 2005. ^5^HIV, human immunodeficiency virus: Lehmann et al., 2005; Do et al., 2014. ^6^VSV, vesicular stomatitis virus: Lehmann et al., 2005. ^7^HHV-8, human herpesvirus-8: Akula et al., 2003. ^8^MARV, Marburg virus: Schudt et al., 2013. ^9^VACV, vaccinia virus: Huang et al., 2008; Mercer et al., 2010b; Taylor et al., 2011.*

## Author Contributions

KC, JB, SH, and VT wrote the article. MV, DS, and VT did the editing work. VT develop the images.

## Conflict of Interest Statement

The authors declare that the research was conducted in the absence of any commercial or financial relationships that could be construed as a potential conflict of interest.
